# Cell-Matrix Interactions and Matricrine Signaling in the Pathogenesis of Vascular Calcification

**DOI:** 10.3389/fcvm.2018.00174

**Published:** 2018-12-07

**Authors:** David Ngai, Marsel Lino, Michelle P. Bendeck

**Affiliations:** ^1^Department of Laboratory Medicine and Pathobiology, University of Toronto, Toronto, ON, Canada; ^2^Ted Rogers Centre for Heart Research, University of Toronto, Toronto, ON, Canada; ^3^Department of Medicine, University of Toronto, Toronto, ON, Canada

**Keywords:** vascular calcification, extracellular matrix, mechanotransduction, collagen, integrin, discoidin domain receptor, cytoskeleton, osteogenesis

## Abstract

Vascular calcification is a complex pathological process occurring in patients with atherosclerosis, type 2 diabetes, and chronic kidney disease. The extracellular matrix, via matricrine-receptor signaling plays important roles in the pathogenesis of calcification. Calcification is mediated by osteochondrocytic-like cells that arise from transdifferentiating vascular smooth muscle cells. Recent advances in our understanding of the plasticity of vascular smooth muscle cell and other cells of mesenchymal origin have furthered our understanding of how these cells transdifferentiate into osteochondrocytic-like cells in response to environmental cues. In the present review, we examine the role of the extracellular matrix in the regulation of cell behavior and differentiation in the context of vascular calcification. In pathological calcification, the extracellular matrix not only provides a scaffold for mineral deposition, but also acts as an active signaling entity. In recent years, extracellular matrix components have been shown to influence cellular signaling through matrix receptors such as the discoidin domain receptor family, integrins, and elastin receptors, all of which can modulate osteochondrocytic differentiation and calcification. Changes in extracellular matrix stiffness and composition are detected by these receptors which in turn modulate downstream signaling pathways and cytoskeletal dynamics, which are critical to osteogenic differentiation. This review will focus on recent literature that highlights the role of cell-matrix interactions and how they influence cellular behavior, and osteochondrocytic transdifferentiation in the pathogenesis of cardiovascular calcification.

## Introduction

Vascular calcification is a pathology characterized by ectopic calcification of the vessel wall of muscular or elastic arteries. Vascular calcification is often observed in atherosclerosis, type 2 diabetes (T2D), chronic kidney disease, and aging, and contributes to increased cardiovascular morbidity and mortality independent of other known risk factors (1–3). Vascular calcification increases the risk of myocardial infarction and heart failure and is the leading cause of the death in patients with chronic kidney disease (4, 5). In addition, patients with high calcium deposition that also have peripheral artery disease have approximately a 25% increase in the risk of limb amputation (6). Despite the widespread incidence of vascular calcification, effective, and targeted therapies are still lacking.

Previously thought to be a passive process of crystal deposition on the surrounding extracellular matrix (ECM), it is now appreciated that vascular calcification pathogenesis is an active process involving the transdifferentiation of vascular smooth muscle cells (VSMCs) into osteochondrocytic cells (7–9). The nucleation and propagation of calcium phosphate crystals in the vessel wall is mediated by matrix vesicles secreted by osteochondrocytic cells deposited on components of the ECM such as collagens and elastin (10). Furthermore, previous studies have found many of the transcriptional and regulatory signaling pathways involved in normal bone formation are also present in calcifying vessels (11–14). Some examples include paracrine signaling molecules like bone morphogenetic protein 2 (BMP-2) (15), the master osteogenic transcription factor runt-related transcription factor 2 (Runx2) (14), expression of pro-calcification enzymes such as alkaline phosphatase (16), and loss of calcification inhibitors such as pyrophosphate (17), matrix GLA protein (18), and fetuin-A (19).

Much research on vascular calcification has highlighted the impact of systemic factors such as inflammation, lipids, glucose, and phosphate on VSMC transdifferentiation. In addition to these factors, it has become evident that the local ECM tissue microenvironment not only acts as a scaffold for cells and hydroxyapatite deposition, but it also plays an important role as a signaling entity, modulator of inflammation, and of cell phenotype. This has been observed in VSMC transdifferentiation to osteochondrocytic cells as well as in other cell types such as mesenchymal stem cells. Matrix binding receptors such as the integrins and discoidin domain receptors (DDRs) have been implicated in the regulation of osteogenic programs. Modulation of cell phenotype by ECM stiffness sensed through matrix binding receptors has also been recognized to be important in osteogenic differentiation. Furthermore, indirect effects of the ECM on vascular calcification may be induced by creating changes in systemic factors or overall metabolism.

In this article, we review the current state of knowledge on the ECM and its role in osteogenesis and vascular calcification at a physiological level, and from a molecular and cellular standpoint. We review mechanisms of ectopic calcification and osteogenic differentiation to provide context for further discussion of matricrine regulation of calcification. We then review ECM proteins and receptors which have been implicated in calcification, in particular DDRs, integrins, the elastin receptor complex (ERC), and receptor for advanced glycation end products (RAGE). Next is a discussion of two important matrix localized calcification inhibitors, osteopontin and matrix Gla proteins (MGP). Finally, compelling evidence for an integrated mechanosensitive matricrine signaling axis involving receptors coupled to the cytoskeleton is discussed. A better understanding of the pathobiology will help to identify potential targets for more effective therapeutics or treatment options.

## Ectopic Calcification

Ectopic calcification is characterized by the pathological deposition of calcium phosphate crystals within the extracellular matrix of soft tissues, including the vasculature (20). Vascular calcification is a form of ectopic mineralization and can be classified as one of two main types: (1) atherosclerotic (intimal) calcification, and (2) medial calcification (21). As suggested by the name, intimal calcification occurs within atherosclerotic plaques, while medial calcification occurs within the medial layer of the vasculature. The cellular mechanisms driving both forms of vascular calcification are similar and share features in common with osteogenic programs (22), however remain distinct in their pathology. Risk factors associated with vascular calcification include chronic kidney disease (CKD) (5, 21, 23), T2D (24, 25), inflammation (26), and age to name a few (27, 28). Medial calcification is more common in patients suffering from T2D and CKD and is thought to be initiated by the nucleation of calcium phosphate crystals in matrix vesicles or on elastin and is exacerbated by metabolic factors such as hyperglycemia and insulin resistance (8). Intimal calcification on the other hand is more closely associated with atherosclerosis and is mediated by local inflammation (8). Both medial and intimal calcification involve the transdifferentiation of VSMCs into osteochondrocytes via mechanisms that are currently being investigated (11, 29–31).

Osteogenic differentiation and bone mineralization are highly regulated processes involving multiple interacting factors including intracellular signaling cascades, secreted factors, extracellular inhibitors of mineralization, and cell-matrix interactions. Several signaling molecules such as fibroblast growth factors (FGFs), transforming growth factors (TGFs), and bone morphogenetic proteins (BMPs) play a role in osteogenic differentiation. The most widely studied are the BMPs, particularly BMP-2. BMPs signal through BMP receptors, transducing downstream signaling through SMAD1/5/8, which in concert with SMAD4 upregulates the expression of the master osteogenic transcription factor, Runx2. Runx2 commits mesenchymal progenitors to the osteoblast lineage and drives osteochondrocytic transdifferentiation and calcification of VSMCs (14, 32). In addition, Runx2 regulates the expression of osteoblast-related genes such as osteocalcin, osterix, and type I collagen (33, 34). Runx2 activity is subject to regulation by phosphorylation events mediated by kinases such as Akt (12, 35), ERK1/2 (36), p38 (37), JNK (36), GSK3β (37), and CDK1 (in response to glucose) (38). Phosphorylation of Runx2 can be activating or inhibitory. Moreover, Runx2 activity is also regulated by nuclear translocation, DNA-binding capacity and interaction with transcriptional co-factors, as well as protection from degradation by binding partners such as Cbfb (39).

Following osteogenic differentiation, calcification can progress by the nucleation and propagation of hydroxyapatite crystals in the ECM. This is mediated by the secretion of matrix vesicles by osteoblasts or osteochondrocytic cells in normal bone formation and pathological calcification, respectively (40, 41). Matrix vesicles are membrane-bound particles of approximately 100 nm in diameter and are a driving force for the initiation and propagation of mineralization. The ECM is an important site for matrix vesicle deposition and scaffolding for biomineralization (41, 42). Secreted matrix vesicles can become deposited and cluster on collagens in the ECM (40, 41). Collagen density has been shown to be negatively correlated to matrix vesicle clustering and biomineralization both in *in vitro* and *in vivo* models of vascular calcification (41).

There are examples of diseases that demonstrate the importance of the ECM in the progression of calcification. Osteogenesis imperfecta is an inherited disease involving a mutation in the α1 or α2 chains of type I collagen leading to reduced deposition of normal collagen fibrils and production of structurally abnormal collagens (43). Improper mineralization of hydroxyapatite crystals on the collagen scaffolding leads to the development of fragile and brittle bones (44). Polymorphisms in the Sp1 binding site of the Col1α1 gene are associated with osteoporosis, a disease resulting in reduced bone mineral density and increased risk of bone fracture (45). This polymorphism increases binding of the Sp1 transcription factor to the Col1α1 gene promoter, causing an increase from the normal 2:1 ratio of Col1α1 to Col1α2 mRNA and protein produced by osteoblasts (46). This may be a causal mechanism for the reduced bone quality and bone mass in osteoporotic patients with this polymorphism.

## Extracellular Matrix

The ECM is important in the regulation of cellular phenotype, homeostasis, and development in addition to providing physical support and organization of cells into tissues and organs (47). Collagens are the main component of the ECM and are made up of α-chains that assume a triple-helical conformation with a repeating Gly-X-Y amino acid motif where X and Y constitute any amino acid (47). Fibrillar collagens are composed of three α-chains, although more than 40 α-chains have been discovered in humans (48). Collagens can form supramolecular structures and can be classified as fibrillar, fibrillar-associated collagens with interrupted triple-helix (FACIT), membrane-associated collagens with interrupted triple-helix (MACIT), long chain, short chain, filamentous, or basement membrane comprised solely of type IV collagen. Although collagens are its main constituent, the ECM also consists of elastin, proteoglycans, lecticans, laminin, and fibronectin (FN). Like collagens, they have unique tertiary structures and contribute to the organization and complexity of the ECM and are reviewed in greater detail elsewhere (47).

The ECM is often referred to as the “matrisome” and consists of over 300 components that have been compiled and reviewed elsewhere (49). The relationship between the ECM and the cells residing within it is a reciprocal one, as matrix binding receptors sense the biochemical and physical makeup of the ECM and transduce signals to the cell which can in turn contribute to ECM remodeling. The major classes of ECM receptors (integrins, DDRs, and ERC) are discussed in further detail in this review.

Another important component of the “matrisome” is matrix bound proteins, such as growth factors that have important functions in the regulation of cell growth, plasticity, and metabolism. ECM components can bind to and sequester growth factors, storing them in a “solid phase” until their release is enabled (50). For example, FN and vitronectin contain known hepatocyte growth factor (HGF) binding sites, and endothelial cell migration was augmented by HGF complexed to FN or vitronectin (51). Similarly, vascular endothelial growth factor (VEGF) was shown to bind tenascin-X (52), and FN (53). FN fragments containing the VEGF binding domain as well as the α_5_β_1_ integrin binding domain were required for the maximal induction of endothelial cell migration and proliferation (53). In some cases, components of the ECM are required for ligand presentation and binding to its receptor. For instance, fibroblast growth factor (FGF) is known to bind to heparin sulfate, a requirement for FGF binding to the FGF receptor (54). Similarly, TGF-β is sequestered in the matrix, and TGF-β ligands are presented by integral membrane proteoglycans (55). In addition to acting as a growth factor reservoir, some ECM components contain growth factor-like domains that activate growth factor receptors directly. Two examples of this are laminins and tenascin, both known to contain EGF domains (56, 57). A hallmark of chondrogenic tissues is type II collagen, which is secreted into the ECM as two splice variants, type IIA and type IIB (58). Chondrocytes secrete mainly type IIB collagen (58), while type IIA collagen is secreted by epithelial and mesenchymal stem cells (59). Type IIA collagen was shown to bind to BMP-2 and TGF-β1, which are important to chondrogenesis and endochondral bone formation (58).

The composition of the ECM is highly dynamic and is regulated by a balance of matrix deposition and degradation by matrix proteases such as the matrix metalloproteinases (MMPs). A total of 23 MMPs have been identified in humans and can collectively degrade all ECM proteins (60). MMPs are secreted as zymogens that require enzymatic cleavage for activation. Endogenous inhibitors of MMPs, tissue inhibitors of metalloproteinases (TIMPs) 1–4, are present to prevent excessive cleavage of matrix components. TIMPs 1, 2, and 4 exist soluble in the extracellular milieu whereas TIMP3 is bound to the ECM (61). Dysregulation of matrix turnover resulting in excessive or insufficient matrix degradation can result in pathologies such as tissue fibrosis and contribute to the production of bioactive signaling molecules signaling through ECM receptors (62, 63). Degradation of matrix components can also result in altered release of growth factors sequestered in the ECM, for example TGF-β (64). Matrix turnover by MMPs is prominent in diseases associated with vascular calcification such as atherosclerosis and T2D, and thus release of pro-osteogenic growth factors may contribute to the pathogenesis of vascular calcification.

Direct signaling through ECM receptors by the matrisome also play an important role in the maintenance of cellular phenotype and function and will be the main focus of this review. Specifically, we will review work on the DDRs, the integrins, the ERC, and the RAGE, and their functions in osteogenesis and vascular calcification. A summary of these receptors, their ligands, and pro-calcific signaling and functions is provided in Table 1.

**Table 1:** Summary of matrix-binding receptors, their respective ligands, and their potential functions in promoting calcification.

**Matrix receptor**	**Ligand(s)**	**Pro-Calcific signaling and function**
DDR1	Collagens I-V, VIII	Akt, ERK1/2, p38, MMP-2/9 activity, maintenance of dynamic microtubules by GSK3β inhibition, stiffness sensing (65–67)
DDR2	Collagens I-III, V, X	Runx2 induction, inhibition of osteoclastogenesis (68, 69)
Elastin Receptor	Elastin-Derived Peptides	Akt, ERK1/2, VSMC proliferation and de-differentiation (70–72)
α_1_β_1_ and α_2_β_1_ integrins	Collagen I, Laminin	Osteoblast attachment, stiffness sensing (73, 74)
α_5_β_1_ and α_v_β_3_ integrins	Fibronectin, RGD-peptide, Osteopontin, Elastokines (α_v_β_3_ integrins)	Stiffness sensing, RGD stimulation enhances VIC and VSMC calcification *in vitro* (75–77)
α_4_β_1_, α_9_β_1_, and α_9_β_4_ integrins	Osteopontin SVVYGLR (SLAYGLR in mice) cryptic motif	Increased immune cell infiltration, increased production of IL-1β, TNF-α, IL-6, IL-17 (78, 79)
RAGE Receptor	Glycated ECM proteins, HMGB1, S100/calgranulin, phosphatidylserine	ERK1/2, p38, JNK, SMAD2/3 activity, cooperation with Nox1 for ROS production, NFκB activation (80–85)

*Listed here are major matrix binding receptors discussed in this review, highlighting their respective ligands as well as known signaling pathways*.

## Discoidin Domain Receptors

There are two DDRs, DDR1 and DDR2, which are collagen binding receptor tyrosine kinases. DDRs have been implicated in cellular processes regulating migration, adhesion, proliferation, as well as in the pathogenesis of fibrosis (86, 87), cancer (88, 89), atherosclerosis (90–92), and vascular calcification (16, 65). The DDRs are activated upon binding to native triple-helical collagens, undergoing autophosphorylation of the cytoplasmic domain which leads to downstream signaling (93). Compared to other receptor tyrosine kinases, the DDRs have delayed phosphorylation kinetics (93). For instance, the epidermal growth factor receptor (EGFR) and the fibroblast growth factor receptor (FGFR) are maximally phosphorylated in a period of seconds to minutes after ligand binding, and are then negatively regulated (94). In contrast, type-I collagen mediated DDR1 phosphorylation peaks at 90 min and is sustained for a period of 18 h (93). In cells grown in suspension, however, DDR1 phosphorylation was accelerated (95), demonstrating a context and adhesion dependent effect on DDR1 phosphorylation and function. Once activated, DDR1 can bind to signaling molecules that include PI3K subunits p85 and p110, STAT-1a/b,−3, and−5b, as well as guanine exchange factors PLC-γ1 and Vav1/2 (96). DDR1 has also been shown to activate P38 (97), ERK1/2 (98), and PI3K/Akt signaling pathways that are important in regulating cellular functions related to proliferation, metabolism and cell differentiation (65, 99).

Recent work from our laboratory has demonstrated that DDR1 promotes vascular calcification in atherosclerosis and diabetes (16, 65). Ahmad et al. studied *Ldlr*^−/−^ mice fed a high-fat diet to stimulate the development of atherosclerotic plaques. DDR1 deficiency resulted in marked reductions in vascular calcification of the atherosclerotic plaques (16). VSMCs harvested from *Ddr1*^−/−^ mice exhibited decreased alkaline phosphatase activity and matrix calcification in *in vitro* calcification assays. We next fed a diabetogenic diet to *Ldlr*^−/−^ mice to induce diabetes and atherosclerosis (65). We found that DDR1 deletion decreased vascular calcification and abolished Runx2 nuclear localization *in vivo*. Cell culture experiments revealed that DDR1 signals via PI3K/Akt and P38 to activate Runx2 leading to VSMC transdifferentiation to an osteochondrocyte-like phenotype. Moreover, we showed that microtubules were required for the translocation of Runx2 to the nucleus, and microtubules were disrupted in DDR1 deficient VSMCs. In contrast to our recent work, a previous study reported that matrix calcification was significantly upregulated in DDR1 deficient VSMCs (100). A probable reason for this discrepancy is the use of different calcification media with a high concentration of β-glycerophosphate compared to our high glucose and phosphate media, because the former media causes cell death and passive calcification which likely obscures late stage phenotypic differences between the cells.

DDR2 has been implicated in chondrogenesis and in cartilage and bone remodeling (68, 69, 101–103). DDR2 is expressed in fibrocartilage within the temporomandibular joint, and deletion of DDR2 resulted in delayed condyle mineralization (104). DDR2 is also involved in bone remodeling. DDR2 overexpression inhibited osteoclast differentiation, and silencing DDR2 enhanced osteoclast differentiation, demonstrating that DDR2 is an important inhibitor of osteoclastogenesis (68). High expression of DDR2 has also been detected in synovial fibroblasts from patients with rheumatoid arthritis. DDR2 overexpression resulted in increased MMP-13 expression dependent on Runx2 and AP1 binding to the MMP-13 promoter (105). Taken together these studies highlight important functions for both DDRs in the regulation of osteogenesis, and specifically for DDR1 in regulating vascular calcification.

## Integrins

Integrins are a family of heterodimeric ECM receptors formed by the dimerization of an α and a β subunit. In humans, 24 distinct heterodimers have been identified with different combinations of 18 α subunits and 8 β subunits (106, 107). Each heterodimer has distinct binding affinities and sequence recognition capabilities to ECM proteins. Upon ligand binding, integrins undergo a conformational change allowing for interaction with downstream intracellular signaling mediators such as focal adhesion kinase (FAK), Rho GTPases, and paxillin. In addition to conformational changes resulting from ligand binding, physical forces can induce structural changes to integrins. Integrins have been implicated in a number of cellular processes such as adhesion, migration, proliferation, and cellular differentiation.

Integrins have a role in osteogenic differentiation and both physiological and pathological calcification. During bone formation, osteoblasts and chondrocytes deposit ECM proteins such as osteopontin and type I collagen which activate integrins. The integrin-ECM interaction guides the progression of bone growth and mineralization. Of the integrins, β_1_ and β_3_ integrins have been studied more extensively. Stimulation of the FN receptor, α_5_β_1_ integrin, in adipose stem cells suppressed osteogenic differentiation (108). Transgenic mice expressing dominant-negative β_1_ integrin in osteoblasts had impaired bone formation, due to impaired adhesion of osteoblasts to the ECM (73). In addition to bone development, integrins have been shown to influence osteochondrocytic transdifferentiation and pathological calcification. Different matrix proteins elicit different effects on *in vitro* VSMC calcification and administration with α_5_-integrin blocking antibodies attenuated FN-mediated enhancement of calcification (75). This is in contrast to observations stated previously on the role of α_5_β_1_ integrin in adipose stem cells and osteogenesis, suggesting a cell type-specific role of α_5_β_1_ integrin in modulating osteogenesis. Work done on valve interstitial cell (VIC) calcification showed that stimulation with the β_3_-integrin ligand Arg-Gly-Asp (RGD) peptide, a motif found in FN, promoted osteochondrocytic differentiation and calcified nodule formation (76). This highlights the importance of integrin signaling to respond to cues from the ECM to modulate osteogenesis and calcification.

## Elastin Receptor Complex

Elastin is a matrix component of large elastic and muscular arteries and is composed of polymerized tropoelastin monomers laid over a scaffold of fibrillin microfibrils, bound to elastin binding, and crosslinking proteins. Primarily synthesized and deposited during fetal development, elastin expression and synthesis of elastin fibers drops off significantly postnatally becoming undetectable by adulthood. Under physiological conditions, turnover of elastin is minimal and the protein has a half-life of several decades (109). In aging or pathological states such as atherosclerosis, elastin fiber integrity is compromised due to degradation by proteases such as MMP-2,−9, and−12 (110, 111), neutrophil elastases (112), and cysteine proteases (113). The proteolysis of elastin leads to the release of bioactive elastin-derived peptides called elastokines. Elastokines signal through cell surface receptors on smooth muscle cells to mediate a wide variety of activities. The elastin receptor complex (ERC) was the first receptor identified and remains the best studied (114), though α_v_β_3_ and α_v_β_5_ integrins (115, 116) as well as galectin-3 (117) can also respond to elastokines.

The ERC is a trimeric protein complex composed of two membrane-bound subunits, protective protein/cathepsin A (PPCA) and neuraminidase-1 (Neu-1), and the elastin-binding subunit, called elastin binding protein (EBP) (118). EBP is responsible for ligand binding through recognizing peptide motifs and Neu-1 is crucial for signal transduction (119, 120). The elastin receptor-activating elastokines include the VGVAPG repeat peptide and GXXPG-containing peptides, where X is any hydrophobic amino acid (119, 121, 122). Negative regulation of elastin receptor signaling is achieved through allosteric inhibition by galactose or lactose, which inhibits EBP binding to elastokines (123). Elastin receptor activation induces multiple intracellular events including activation of tyrosine kinases such as FAK and c-Src converging on activation of ERK1/2 and Akt (70–72).

Proteolytic degradation of elastin and signaling by elastokines and tropoelastin has been implicated in the progression of vascular calcification. Initial observations were made correlating the expression of MMP-2 and MMP-9 with regions of calcified elastin and osteogenic differentiation of VSMCs *in vivo* (124). More recent studies have shown that elastin breaks colocalize with regions of calcification in the aortas of Marfan syndrome patients (125). Treatment of VSMCs with elastokines promoted osteogenic differentiation and calcification *in vitro* and this was further enhanced with concomitant TGF-β treatment (126), or culture in high-glucose or high-phosphate media (127, 128).

Studies in mice with chemical or genetic inhibition of proteases have demonstrated that elastin degradation contributes to calcification. One of the earliest studies to directly demonstrate the effect of elastolysis on vascular calcification was done using a rat model of abdominal aortic aneurysm induced by low dose CaCl_2_ treatment (129). This protocol induced elastin breaks by increasing MMP-2 and MMP-9 activity, and tissues were harvested prior to the development of inflammatory response and alterations in vessel morphology due to abnormal hemodynamics. CaCl_2_ treatment caused calcification of the elastic fibers where breaks had been induced (129). Furthermore, MMP-2/MMP-9 deficiency or treatment with AlCl_3_, an inhibitor of MMP-2/9, attenuated degradation of elastic fibers and calcification (129). In other studies, the MMP inhibitor doxycycline was used to inhibit elastase activity in vitamin D_3_ or warfarin-induced rat models of vascular calcification and it was shown that this significantly inhibited vascular calcification (130–132). Nephrectomy is a commonly used model of chronic kidney disease which results in vascular calcification. Knockout of the elastolytic protease, cathepsin S (catS), in nephrectomised *ApoE*^−/−^ mice attenuates elastin degradation and vascular and valvular calcification (133). Analysis of the blood biochemistry of *ApoE*^−/−^*; CatS*^−/−^ mice following nephrectomy revealed reduced Cystatin C (marker for dysfunctional glomerular filtration), increased cholesterol, and similar phosphate and calcium concentrations compared to *ApoE*^−/−^*; CatS*^+/+^ mice following nephrectomy (133). Furthermore, histological analysis of carotid artery plaques from these two groups of mice revealed similar macrophage content as assessed by Mac-3 staining (133). Administration of cathepsin S to VSMCs treated with elastin peptides in culture enhanced *in vitro* calcification (133). TGF-β is normally sequestered in the ECM by the matrix-bound large latent complex (LLC) until released by matrix degrading proteases during matrix remodeling. The latent TGF-β binding proteins (LTBPs) are key components of the LLC and tether to elastin scaffold proteins and elastin crosslinking proteins such as fibrillin-1 and fibulin-5 (134, 135). Given these findings, proteolytic degradation of elastin and release of TGF-β may be a contributing mechanism to the development of vascular calcification. TGF-β is a known mediator of fibrosis and has been shown to stimulate VSMC calcification. Interestingly, calcifying vessels in mice lacking MGP had enhanced expression of elastin and abnormal elastin structure in the medial layer compared to non-calcifying wild-type mice (136). Furthermore, elastin haploinsufficiency (*Eln*^+/−^) on the MGP-null background reduced spontaneous vascular calcification (136). These results suggest that the quantity of elastin produced influences calcification, possibly through the scaffolding role of elastin for hydroxyapatite deposition or availability of elastin for the generation of elastokines which influence osteochondrocytic transdifferentiation.

## Receptor for Advanced Glycation End Products (RAGE)

Vascular calcification is a major complication in T2D. One of the prominent hallmarks of T2D is hyperglycemia. Excessive circulating glucose can result in non-enzymatic glycation of proteins, altering their structural and functional properties. Glycation is a chemical modification by which a sugar moiety covalently modifies amino acid residues on proteins. Given the relatively long half-life and length of ECM proteins such as collagens, they are one of the major protein groups that are glycated in diabetes (137). Glycation of collagens can lead to structural deficits resulting in impairment of collagen cross-linking (138), increased matrix and tissue stiffness (139), inhibition of matrix turnover (140), defective cell-matrix interactions (141), and reduced sliding of collagen fibrils (142, 143). In addition to structural changes, glycated proteins can serve as ligands for the multi-ligand RAGE (144). RAGE are cell surface receptors found on most immune cells as well as on other cell types such as VSMCs (145–148). Aside from advanced glycation end products (AGEs), other ligands have been identified for RAGE such as the secreted proteins S-100/calgranulin (80) and high mobility group box 1 (HMGB1) (149), as well as phosphatidylserine (150). Ligands like S100/calgranulin family members can act through RAGE to promote the inflammatory NFκB pathway as well as ROS signaling through cooperation with NADPH oxidase-1 (Nox1) upon ligand binding (80, 81, 151). Downstream kinase mediators such as ERK1/2, p38, and JNK as well as TGF-β pathways are also activated by RAGE stimulation (82–85). A soluble RAGE is secreted by cells to act as a decoy receptor to attenuate RAGE signaling (149).

Recent studies have identified an important role for membrane RAGE and Nox1 signaling in VSMC osteochondrocytic differentiation and vascular calcification (81, 152). RAGE is upregulated in calcified tissues in rat models of diabetic aortic medial calcification as well as aortic valve calcification in *ApoE*^−/−^ mice (153, 154). NFκB can increase the expression of RAGE, thus providing a positive feedback mechanism for this pathway (155, 156). In the assessment of human diabetic patients following foot amputation, it was found that levels of circulating AGEs and expression of RAGE in the anterior tibial artery wall was positively correlated with the extent of calcification (157). Circulating levels of the inhibitory soluble RAGE were reduced in patients with calcific aortic valve stenosis and with vascular calcification (158, 159). *Ex vivo* stimulation of diabetic rat femoral arteries with the RAGE ligand N-methylpyridinium enhanced calcification (160). Treatment of human VSMCs with AGEs from the serum of diabetic patients upregulated the expression of alkaline phosphatase, Runx2, and other osteochondrocytic proteins (161). On the other hand, RAGE-blocking antibodies, p38 inhibition, and Nox inhibition prevented calcification and VSMC osteochondrocytic transdifferentiation (81, 83). *In vivo* studies in *ApoE*^−/−^ mice fed a high cholesterol diet showed that RAGE deficiency attenuated valvular calcification, and this was associated with reduced ER stress and inflammation without changes in lipid profile (154).

## Osteopontin

Osteopontin is a matrix protein secreted by osteoblasts, macrophages, smooth muscle cells, and chondrocytes. This protein upon its secretion can integrate into the matrix through its negatively charged amino acid residues. Furthermore, it possesses a RGD sequence allowing for its recognition by integrin heterodimers including the α_v_β_3_ integrin. Osteopontin also contains a distinct binding site for CD44 on immune cells which promotes cell adhesion and migration. Osteopontin contains a cleavage site for thrombin, which upon cleavage reveals a cryptic site for recognition and signal transduction by alternative integrin heterodimers α_4_β_1_, α_9_β_1_, and α_9_β_4_ (162–164). Recognition of the cryptic site by integrins enhances immune cell migration and promotes inflammation (78, 79). Bone marrow derived macrophages likely recognize osteopontin primarily through the cryptic site since flow cytometry has identified that ~95% are positive for α_4_ and α_9_ integrins, but only ~5% for α_v_ integrins (165).

Osteopontin has a strong affinity for calcium allowing it to interact with hydroxyapatite (166). It has been identified to have a role in inhibiting biomineralization and negatively regulating calcium crystal formation both in physiological bone formation (167) and pathological ectopic calcification (168). Osteopontin also has roles modulating the formation and function of bone remodeling cells such as osteoclasts. Osteopontin stabilizes osteoclasts on the bone as it facilitates resorption and promotes the survival of osteoclasts (169–171). Osteopontin is upregulated in calcified vessels and possesses an inhibitory role in vascular calcification (172). Furthermore, circulating osteopontin has been identified as a biomarker for vascular calcification in diabetic patients (173). Knockout of osteopontin in spontaneously calcifying MGP-deficient mice or mice fed a high-phosphate diet accelerated the development of vascular calcification and death (174, 175). *In vitro* calcification of cultured VSMCs was found to be exacerbated by genetic deletion of osteopontin and prevented by reintroducing the osteopontin gene by retroviral transfection (176). Furthermore, loss of osteopontin promotes apoptosis of VSMCs, which may contribute to enhanced calcification (177). These studies implicate a beneficial effect of osteopontin in slowing the progression of vascular calcification. Interestingly, human peripheral monocytes and macrophages from hypertensive patients with vascular calcification had reduced potential to form osteoclasts following osteopontin stimulation (178). This indicates that osteopontin has a suppressive role for the turnover of hydroxyapatite in this context.

In addition to its effects on biomineralization, osteopontin has numerous roles in regulating smooth muscle cell phenotype and inflammation. Osteopontin promotes smooth muscle cell migration (179) and phenotypic switching by downregulating contractile markers and increasing proliferation (180, 181). Osteopontin is expressed widely by immune cells such as macrophages and is upregulated during the process of inflammation and wound healing (182, 183). It can facilitate differentiation of monocytes and allow adhesion and migration of immune cells through the engagement of adhesion receptors including integrins and CD44 (184–186). The osteopontin promoter contains pro-inflammatory response elements such as AP-1-binding sites and NFκB binding sites (187, 188). Stimulation of macrophages with LPS and pro-inflammatory cytokines such as IL-1β induces the expression of osteopontin (189). Osteopontin knockout in *ApoE*^−/−^ mice attenuated macrophage infiltration into plaques and MMP-2/9 activity, resulting in a reduction in atherogenesis (190). Stimulation of monocytes/macrophages from hypertensive patients with vascular calcification with recombinant osteopontin reduced inflammatory cytokine secretion and increased anti-inflammatory cytokine production (178). However, osteopontin had no effect on monocytes/macrophages from hypertensive patients without vascular calcification (178). This finding of an anti-inflammatory effect contrasts with the findings stated previously. However, it may suggest a context-dependent response by monocytes/macrophages to osteopontin and through the influence of other factors in the calcifying environment.

## Matrix Gla Protein (MGP)

MGP is a vitamin K-dependent, secreted matrix protein expressed by all tissues and multiple cell types including VSMCs, macrophages, and osteoblasts. MGP was first isolated in bone and was found to accumulate significantly in the ECM of bone compared to non-calcifying tissues such as the kidney, lungs, and heart (191). The mRNA expression of MGP in osteoblasts, however, was lower than that of the non-calcifying tissues (191). MGP was implicated as an inhibitor of vascular calcification following a study showing that its deletion in mice led to spontaneous vascular calcification and death within 2 months from arterial rupture (192). MGP possesses anti-calcific effects through inhibition of hydroxyapatite mineral formation (193) and BMP signaling (194, 195). Examining the expression pattern of MGP in human vessels, normal vessels exhibit a gradient with high levels of MGP on the luminal side that decreases gradually toward the medial layer (196). VSMCs normally express MGP and high levels of MGP can be found in the fibrous cap of atherosclerotic lesions (196). A negative correlation was found with areas of low or absent MGP expression in the vessel with calcification and Runx2 expression for both intimal and medial calcification (196). MGP expression is downregulated in calcified atherosclerotic human arteries compared to non-calcified vessels (196). *In vitro* calcification studies done with bovine VSMCs found reduced MGP mRNA expression in calcifying cells and had an inverse correlation with the extent of calcification (197). Inhibition of calcification with bisphosphonates restored the expression of MGP mRNA in VSMCs cultured in calcifying media, suggesting that the process of calcification precedes the downregulation of MGP (197). Some conflicting evidence was found, however, showing MGP mRNA was upregulated in calcifying human VSMCs *in vitro* (198). This is likely a result of species-specific differences or time-dependent fluctuations in the expression of MGP. For MGP activity, the protein must be gamma-carboxylated in a vitamin K-dependent manner (199). The anti-coagulation medication, warfarin, is known to be a vitamin K antagonist. A previous study has found that 2 weeks of warfarin treatment in young rats induced significant focal vascular calcification (200). Isolated VSMCs treated with warfarin have reduced carboxylated MGP and increased *in vitro* calcification (201). This was shown to be vitamin K-dependent as the reintroduction of vitamin K to warfarin-treated rats could inhibit calcification (201, 202).

## Matrix Stiffness Sensing

The stiffness of the matrix is measured by how much force is necessary to deform a substrate. Stiffness is often measured as an elastic modulus in kilopascals (kPa). The elastic modulus is defined as the ratio of the stress, which is the force of deformation divided by the area to which it is applied, to the strain, which is the ratio describing the relative deformation compared to the object's original state. The composition, density, and structural integrity of the extracellular matrix play a significant role in determining the stiffness of a tissue. Increased density of collagens such as type I collagen and the glycoprotein FN enhance stiffness whereas glycosaminoglycans such as hyaluronic acid permit greater compliance of the ECM. Increased matrix degradation by MMPs reduces matrix stiffness, whereas increased crosslinking of matrix proteins by lysyl oxidase (LOX) and glycation of matrix proteins enhance matrix stiffness.

**Figure 1:**
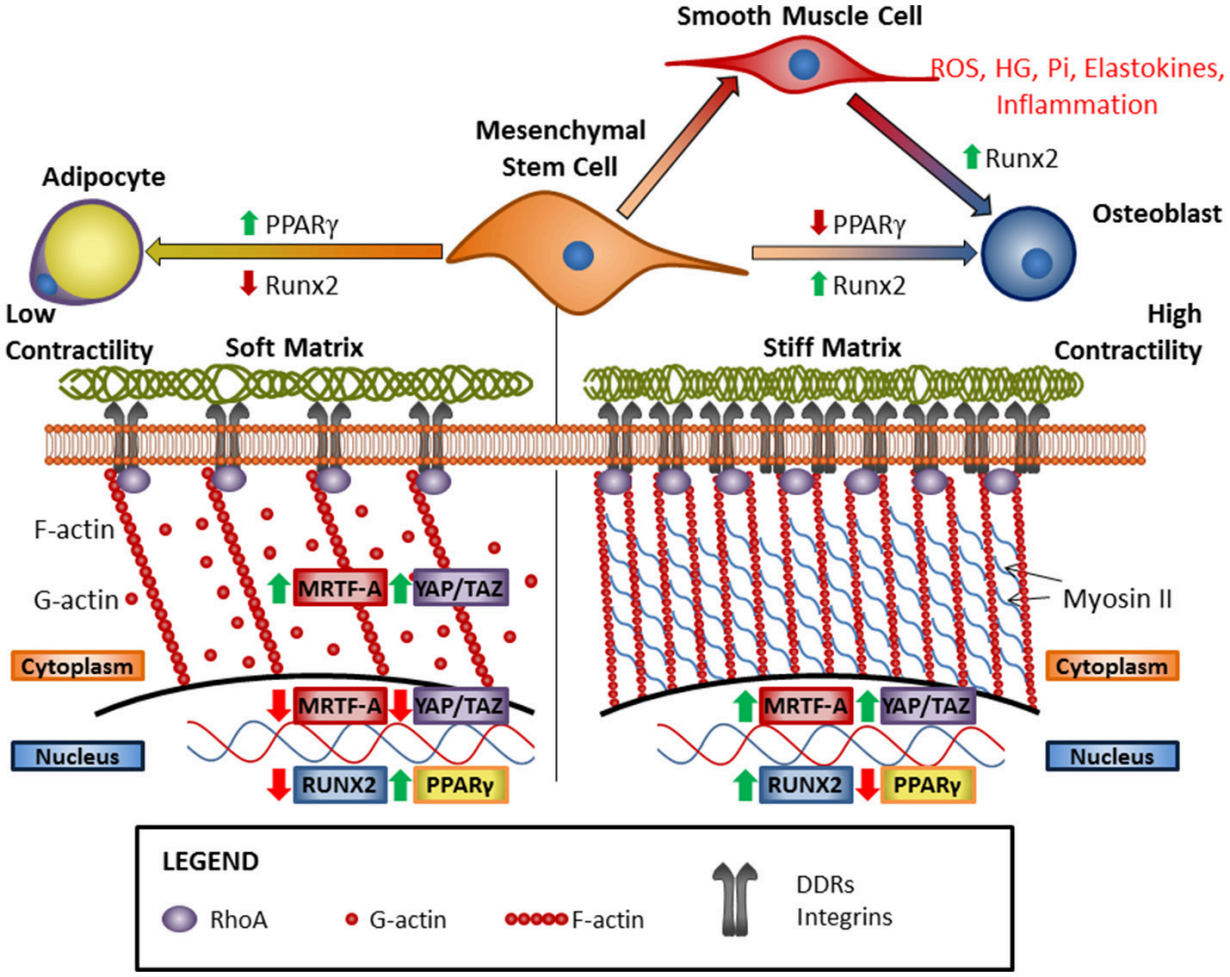
Increasing matrix stiffness drives osteogenic differentiation via cooperative ECM receptor signaling and modulation of cytoskeletal dynamics. Classically, matrix stiffness is known to be sensed by integrins, but recent research has shown that DDR1 can also act as a mechanosensory receptor and is an important mediator of vascular calcification. Vascular calcification occurs as VSMCs transdifferentiate into osteoblast-like cells and can be promoted by RUNX2, high-glucose (HG), inorganic phosphate (Pi), elastokines, and inflammation. Increased matrix stiffness leads to osteogenic differentiation of mesenchymal stem cells and the cytoskeleton plays an integral role in this process. Stress fiber formation due to increased RhoA activity results in increased nuclear internalization of fibrogenic/myogenic transcription co-factors MRTF-A and YAP/TAZ, and osteogenic transcription factor Runx2, and the concomitant inhibition of adipogenic transcription factor PPARγ. Conversely, reduced ECM stiffness leads to reductions in ECM receptor activation and stress fiber formation, leading to nuclear exclusion of MRTF-A and YAP/TAZ, and reduced Runx2 activity. The resulting increase in PPARγ activity results in adipogenic differentiation.

In addition to biochemical and chemical mediators, cells experience a variety of physical cues that influence their behavior, phenotype, and differentiation. By a process called mechanotransduction, cells can convert physical cues or forces into biochemical signals affecting cellular responses. The stiffness of the matrix is detected by matrix-binding cell surface receptors including the integrin family of receptors and DDRs. The mechanism by which this occurs is illustrated schematically in Figure 1. Matrix receptors sense the stiffness of the ECM by binding and tugging on the ECM via their extracellular domains and interacting with signaling molecules and the cytoskeleton via their cytosolic domains. Resistance or stiffness of the ECM triggers the reorganization of the cytoskeleton, polymerization of actin monomers (G-actin) into filamentous actin (F-actin) and bundling of F-actin into contractile units known as stress fibers. Cells contract in response to external forces, with myosin generating tensional forces on the actin fibers increasing the stiffness of the cytoskeleton (203, 204). Intact microtubules are also necessary for cells to respond to externally applied forces (204). Tension forces generated by actin stress fibers deform the nuclear envelope through their interaction with the LINC (linkers of the nucleoskeleton to the cytoskeleton) complexes (205). Increased tension on the nuclear envelope promotes the expression and reduces degradation of an abundant structural protein in the nuclear envelope, Lamin A (206). This increases the tensile strength of the nuclear envelope when cytoskeletal tension is high.

Since collagens and FN are abundant in the ECM, it is not surprising that integrins binding these molecules play important roles in mechanotransduction. Numerous studies have demonstrated the role of type I collagen-binding α_1_β_1_ and α_2_β_1_ integrin heterodimers (74, 207, 208), and FN-binding α_5_β_1_ and α_v_β_3_ integrin heterodimers (77, 209) in mechanotransduction for a variety of cell types including mesenchymal stem cells, fibroblasts, and HEK293 cells. In more recent years, Ghosh et al. showed that DDR1 was a mechanotransducer in adipose-derived stem cells (ASCs) (66). In ASCs, increased compliance of the substrate and reduced cell contractility promoted aromatase and estrogen expression. Knocking down DDR1 with siRNAs attenuated aromatase expression on a soft substrate without influencing stress fiber formation, suggesting that DDR1 helps cells sense matrix compliance (66). Coelho et al. have shown that DDR1 activation is enhanced on type I collagen-coated stiff substrates compared to soft substrates, and that interaction of DDR1 with non-muscle myosin IIA triggers cell contraction and reorganization of the fibrillar collagen ECM (210). Furthermore, *in vivo* studies of rat dermal wound healing found increased DDR1 expression and activation in myofibroblasts when mechanical force was applied to the wound by splinting (210). DDR2 expression has been shown to increase with increasing matrix stiffness (211) and DDR2 has been implicated in cell shape changes in response to changes in matrix stiffness (212). However, evidence is still lacking to show whether or not this receptor is a mediator of mechanosensing.

## Matrix Stiffness and Osteogenesis

A seminal paper published by Engler and colleagues showed that mesenchymal stem cell fate was determined by substrate stiffness (32). Osteogenesis was favored and promoted on a type I collagen-coated substrate with a stiffness of 34 kPa, which is within the range of stiffnesses of collagenous pre-calcified bone (25–40 kPa). Furthermore, pharmacological inhibition of non-muscle myosin IIa by blebbistatin blocked differentiation of mesenchymal stem cells. This highlights the importance of stiffness sensing in osteogenic cell differentiation.

Three transcription factors are responsive to changes in matrix stiffness, and they play roles in osteoblast differentiation, smooth muscle differentiation, and vascular smooth muscle calcification. These are the Yes-associated protein (YAP) and its related protein Tafazzin (TAZ) (213), and Myocardin related transcription factor-A (MRTF-A) (214). All three depend on actin polymerization for the regulation of cytoplasmic and nuclear localization and therefore activity (215–217) (illustrated schematically in Figure 1). MRTF-A is sequestered in the cytoplasm by binding to G-actin but is released to translocate to the nucleus upon actin polymerization to F-actin in cells under mechanical stress (215). In a similar mechanism, YAP/TAZ binds to angiomotin (AMOT) in the cytoplasm until G-actin polymerizes to F-actin, which binds AMOT and allows YAP/TAZ to translocate to the nucleus (218). Actin polymerization also inhibits the Hippo pathway, which is the canonical pathway that mediates YAP/TAZ phosphorylation and prevents its nuclear localization (216). In addition, mechanical stress can induce deformation of the nucleus allowing YAP to be transported into the nucleus through nuclear pores (219). In mechanotransduction, actin polymerization is able to regulate YAP/TAZ nuclear localization independently of canonical Hippo pathway signaling (217). This establishes YAP/TAZ as a mechanosensitive transcriptional co-factor, although further studies are necessary to understand the mechanisms at work.

In mesenchymal stem cells, TAZ forms an activating complex with Runx2 to promote osteogenesis and calcification while suppressing peroxisome proliferator-activated receptor-γ (PPARγ) -mediated adipogenesis (33, 220). YAP/TAZ can influence vascular calcification by enhancing phenotypic switching of VSMCs, or by promoting the inflammatory response. YAP blocks serum response factor (SRF) transcriptional activity to facilitate the conversion of VSMCs from the contractile to the synthetic phenotype, a process which precedes vascular calcification (221, 222). In addition, YAP/TAZ is upregulated in endothelial cells exposed to pro-atherogenic oscillatory blood flow and stimulates the inflammatory response which exacerbates vascular calcification (223, 224).

MRTF-A is an important transcriptional co-factor for SRF, the master regulator of VSMC specific gene expression (225). MRTF-A has also been implicated in osteogenic differentiation of mesenchymal stem cells, and in the transdifferentiation of heart VICs and VSMCs during pathological calcification. MRTF-A KO mice have defects in osteogenesis (226). In human aortic valve fibrosis and calcification, there is upregulation of MRTF-A and smooth muscle α-actin expression in calcified regions, concurrent with differentiation from the VIC to the myofibroblast phenotype (227). In VSMCs grown in normal media, MRTF-A acts downstream of BMPs to maintain the differentiated smooth muscle phenotype (228). However, under calcifying conditions, Runx2 interferes with MRTF-A/SRF to downregulate VSMC-specific genes and upregulate osteochondrocytic genes (229). Since both MRTF-A and YAP/TAZ are regulated by stiffness and actin dynamics, there is extensive crosstalk between the pathways which can be cooperative or mutually inhibitory (230–232). Furthermore, the net response from their signaling is modulated by activation of TGF-β-Smad3 (230). Excessive matrix turnover in vascular pathologies may result in increased release of the pro-fibrotic TGF-β, thus increasing matrix stiffness and cooperative signaling with MRTF-A or YAP/TAZ in VSMCs to enhance osteochondrocytic differentiation.

## Cytoskeleton and Nucleoskeleton in Osteogenesis: Roles of Tubulin and Lamin A

The actin and microtubule cytoskeletons play important roles in mechanotransduction (233), transport of vesicles and proteins (234), and are involved in mediating osteogenesis (235–238). Microtubules can participate in signal cascades by affecting the distribution and compartmentalization of signaling molecules and transcription factors. Recently, we have identified DDR1 as an important mediator of vascular calcification, acting in part to maintain an intact microtubule cytoskeleton which allows Runx2 translocation to the nucleus (65). Studies in tumor cells have demonstrated that Runx2 binds to α-tubulin via its amino terminus, and that these interactions with tubulin are necessary for the nuclear export of Runx2 and sequestration of the transcription factor in the cytoplasm in these cells (239). Interestingly, a microtubule-associated protein, doublecortin-like and CAM kinase-like 1 (DCAMKL1), has been identified as a regulator of osteogenesis. DCAMKL1 blunted osteogenesis by inhibiting Runx2 and genetic disruption of DCAMKL1 in mice resulted in increased bone mass (240). These studies support the notion that microtubules affect osteogenesis by regulating the intracellular localization of Runx2, however the precise mechanisms are complex and vary between different cell types.

Lamin A is a mechanosensitive nuclear envelope protein, a link between the cytoplasmic and nuclear cytoskeleton, and can form complexes with transcription factors. Lamin A is an important reservoir and regulator of transcription factors and co-factors involved in cell fate specification for cells in the mesenchymal lineage, and likely acts by several overlapping mechanisms (241). Lamin A regulates YAP and SRF transcriptional activity by promoting YAP nuclear localization and regulating nuclear actin dynamics to promote SRF activity (206). Lamin A also binds to and promotes Runx2 activity and calcification in VSMCs, osteoblasts, and mesenchymal stem cells, facilitating nuclear transport of Runx2 and enhancing its DNA-binding and transcriptional activity (242, 243). Lamin A is upregulated in rat VSMCs cultured in high calcium and phosphate calcifying media (242). The unprocessed Lamin A precursor, prelamin A, accumulates in calcifying and senescent VSMCs *in vitro* and promotes the osteogenic transcriptional program (244). Increased accumulation of prelamin A in calcifying vascular cells was also observed *in vivo* in child dialysis patients with medial calcification (244).

## Conclusion

Our understanding of the pathology of vascular calcification has advanced considerably, and it is now understood to be an active process driven by VSMC transdifferentiation into osteochondrocyte-like cells. There has been increased interest in understanding how cell-matrix interactions influence cellular responses during vascular calcification. The ECM is constantly changing in normal physiology and in pathology, and its role extends beyond that of a scaffold to that of a signaling mediator with important effects on the transcriptional regulation of cell phenotype. Further investigation into matricrine regulation of cell phenotypes will lead to the development of novel therapeutics to prevent or reverse vascular calcification.

## Author Contributions

MB, DN, and ML conceived of the ideas and outline for the manuscript. DN and ML wrote the first drafts, and MB made suggestions for improvements and additions, and edited the manuscript.

### Conflict of Interest Statement

The authors declare that the research was conducted in the absence of any commercial or financial relationships that could be construed as a potential conflict of interest.
